# Payment of the Medical Staff

**Published:** 1918-03-23

**Authors:** 


### THE BRITISH HOSPITALS ASSOCIATION.

### PAYMENT OF THE MEDICAL STAFF.

At the conclusion of the formal business before the annual meeting of the British Hospitals Association on March 15 (the report of which we hope to publish next week), and the adoption of the report, a discussion was instituted by representatives of some smaller hospitals.

**Mr. Francis Pentey,** York County Hospital, had been deputed to ask whether provincial hospitals were represented when settling terms of allowances during the war, and, if so, by what number. His hospital considered the terms to be totally inadequate. Representatives from endowed London hospitals should not have been allowed to settle this matter without consulting the less endowed provincial hospitals.

**Mr. Wade Deacon** replied that Mr. Cronshaw, representing the Radcliffe Infirmary, Oxford, and he (the speaker), representing the Royal Infirmary, Liverpool, and two other provincial members were present.

**Dr. Neal** (British Medical Association) said he felt sure the action taken by the Medical Officers of the York Hospital was very exceptional. The scheme agreed upon by this Association was carefully considered by the British Medical Association, and approved by them. Moreover, reports being received by the latter body from all parts of the country showed that the hospitals generally were approving the agreement, and taking action to accord with it. It was fully recognised that the proposed remuneration to the medical staffs was quite inadequate, but the principle that some remuneration was due to them for the services they were rendering these patients was secured.

**Mr. Cook** (Cheshire) said that at the small hospital he represented they were receiving two guineas a week for each patient, half of which was paid to the medical man. He thought medical men should be paid a reasonable sum for the time and consideration given. The circumstances connected with large London and provincial hospitals were different from those of the smaller ones, because many of the latter had not a medical staff at all, but the medical men were paid for their attendance on the patients.

**Lord Sandhurst** asked speakers to remember that this proposal was particularly stated to be for one year. That being so, if there were institutions which felt discontented or critical concerning the arrangement, it would be fully within their duty to send a report of their case to this Association, whose Council would give it every care and consideration.

**Lord Knutsford** said the representative from York seemed to be under the impression that his hospital was compelled to accept the new terms. It was within the right of any hospital to say the terms were not adequate, and therefore they would not accept them. But they were the best that could be got out of the Pensions Committee. The services of no medical staff of a hospital could be adequately paid; the days for any such idea had gone by. But, as Dr. Neal said, this recognised the principle of the great services which the profession rendered in hospitals. At the London Hospital it was intended to offer the medical staff 10 per cent. of the contribution from the Ministry of Pensions, but if the physicians and surgeons did not like it they need not have it. They had been attending patients all along for nothing, and he had always been taught that something was better than nothing. The medical staff at the London could, if they wished, go on treating patients for nothing, and give him the balance for the hospital. (Laughter.)

### THE PROPOSED MINISTRY OF HEALTH.

**The Hon. Sir Arthur Stanley, G.B.E., M.P.,** then introduced this subject for discussion and how it may affect the voluntary hospitals. He said he was so anxious to hear the views of the various gentlemen present that he did not propose to introduce the discussion by means of a long speech. It was always easy to arrange a meeting of the London representatives, and therefore he particularly wished to elicit views from representatives of provincial hospitals on this occasion. The speeches of Mr. Pentey and of Mr. Cook showed how difficult were all the problems with which hospitals were confronted. He did not feel qualified, especially in the presence of experts in hospital management, to express an opinion as to how the proposed Ministry of Health would affect the hospitals: in the first place, there was as yet no Bill, so that it was not known what was intended. But what he wished to strongly urge upon the Association was the absolute necessity of having a first-rate Committee sitting in London, or at least one able to be convened at short notice, having on it representatives from various parts of the country, to watch the Bill, when it had been produced, through every one of its stages. Every stage of such a measure would present questions the settlement of which was bound to vitally affect the future, even the very existence, of the voluntary hospitals of the country. When he began to have anything to do with these matters—which was at the outset of the war, when he took up Red Cross work—he found that so far as the Local Government Board was concerned there was an admirable institution, the Association of Poor-Law Unions, which was able to speak in the name of every infirmary in the country, so that when one was dealing with any matter affecting those institutions through that body, one felt that the whole of the infirmaries' interests were being voiced. Serious difficulties had arisen from the fact that the voluntary hospitals of the country had no such body with that complete representation. Within the last few months this Association had taken up questions with the Government such as that discussed a few minutes ago, and, with all due deference to two speakers, they had done it most ably. Whether the arrangement arrived at was satisfactory remained to be seen. It was, however, tentative, for it was difficult to arrive at what price the Government ought to pay. For the Red Cross hospitals a capitation grant of 3s. was obtained. Some said they could not afford to do it on that: others did so. At a meeting called to consider the question he urged that 4s. should be applied for, but the majority favoured 3s. 6d. In the result, as might have been expected from a Government Department, they got a little less, namely, 3s. 3d. A vast number of hospitals in the country were dealing with the same class of patients on a grant of 3s. 3d. and finding it enough. It was easy to understand why: those hospitals had not got the same head charges and other expenses always going on as had the great hospital over which Lord Knutsford presided, and others. The question arose as to how far it was right to put any part of that burden on the Government seeing that the charges would go on, whether there were any military and naval patients in the institution or not. The point illustrated how difficult it was to settle these matters for all hospitals on a uniform plan. If Lord Knutsford had not spoken, he (Sir Arthur) had intended to say that the hospital beds would be filled anyhow, even if there were not these military cases in them, and filled with cases for which the hospitals would receive nothing. Hence, according to the speech of the gentleman from York, they were about 14s. to the good. If it was to be admitted that the Government was to step in and to pay full remuneration to the medical staff, that, after all, was a step towards the Government providing the maintenance generally, and that would at once be admitting the whole principle of Government control. And that, he felt sure it was the opinion of every one in this room, would be nothing short of a disaster to the country, *i.e.*, if the voluntary hospitals were to disappear. Some hospitals were fortunate in having a fairly substantial endowment. Others seemed to be able to get money whenever they wanted it. But he felt very strongly that if the spirit of voluntary help to the sick and wounded were killed, the nation would have lost a very valuable national asset. But he did not think the country would allow it to be killed.

He felt certain a Ministry of Health would come, and he, personally, welcomed it—he was not now speaking for St. Thomas's Hospital. When a Bill was produced, many questions would be raised, and he did not know in what form. And that brought him back to the point with which he commenced, that he hoped the Association would take this matter into consideration, and appoint a small Standing Committee to watch every stage of the Bill when it should appear. On that subject it would be most helpful to hear the views of those who had been good enough to attend to-day.

**Lord Knutsford** said he thought it might be helpful if he were to tell the meeting what was the proposal which the Government had practically undertaken to incorporate into a Bill to provide for a Ministry of Health. A Local Government Committee had made a report to the Minister of Reconstruction, and the lines were suggested on which the Bill should be drawn. The scheme included a provision for all Boards of Guardians to be abolished, and all the rate-paid work which those bodies did, their work for the sick poor (including lunatics), handed over to the County Councils or Borough Councils. The voluntary hospitals were not referred to: they were left as at present. The voluntary hospitals would be asked to enter into contractual arrangements with the Public Health Authority. Where the Borough or County Council could not provide out of the rates what a patient wanted, they would enter into contracts with voluntary hospitals to supply them, a practice at present carried out in regard to cases of ringworm, adenoids, aural disease, and eye troubles. However proud any of those present might be of the voluntary hospitals, they could not contend that they were really meeting the needs of the nation, but people were treated in them when they got serious illnesses. They were not following up that treatment into the patients' homes: there was not the needed continuity of treatment. And the cost of what the hospitals did fell on the few, not on the many; moreover, the revenue of hospitals was always too fitful. Neither would the hospitals meet the needs of the nation in the future, but they would be able to supply what the ratepayers never would supply. The real need for voluntary hospitals would thus be clearly seen: they had invention, they had initiation, ability, and willingness to lavish money on a new invention which gave promise of benefit. If, for instance, someone were to discover a new lamp for the treatment of lupus, it would at once be adopted by a voluntary hospital and tried. But one would not be able to get people elected by the ratepayers to try anything new: they would feel themselves bound by statute, and by the demand that no experimentation should be carried out with ratepayers' money. Even if voluntary hospitals were to be taken over by the State, someone would soon start a special hospital for something or other, and it would be followed by others. As a matter of fact, there would never be an end of voluntary effort. He felt sure there would be no attempt, in the new Bill, to interfere with voluntary hospitals. He hoped, therefore, there would be no pig-headed opposition to a Bill which was going to try to adequately treat the health of the whole nation: let this Association give the Government in this matter all the loyal assistance it could, by its skill, its knowledge, and its inventions. If, in return for hospitals entering into contracts with County Councils, a certain amount of representation was asked for, do not let them be frightened and regard it as State control. Had any of his hearers found that interference meant control? He had never done so: he was only too thankful when people would interfere if they thought they could do the work better than one could do it one's self—and that was quite easy. Therefore he urged the representatives of voluntary hospitals to welcome this Bill, and to do all they could to help it forward.

**Mr. H. J. Toulmin** (St. Albans) thought there was a tendency nowadays to centralise a great deal too much. There should be more local opinion and local administration.

**Major McAdam Eccles, M.S.,** thought all those present would join Lord Knutsford in welcoming a Ministry of Health: he believed the whole medical profession would do so, always provided that profession had a real say in the matter of its institution and its government. Voluntary hospitals not only should, but must, continue as such. The reason for that feeling was that they were British and had a sentiment within them which had probably been handed down from generation to generation—that there was such a thing as charity in its truest and best sense. He had seen State hospitals and their work in Germany, and all he would say was that he sincerely trusted that when we had achieved the victory we should not copy the German principle of State hospitals, for that principle excluded that one human touch which was the very backbone of the voluntary hospital system. A question which was increasing with each day was that of, not State aid, but State payment for services rendered. If patients were taken under the wing of the State, it was the duty of the State not only to see that they were adequately treated, but that some payment should be made for the treatment. There was plenty of evidence that the work done by voluntary hospitals had official recognition, and members of the Association should settle exactly what position they intended to hold in this matter: they should be definitely ready against the time that the proposed Bill came forward. Hence he regarded the proposal to appoint a Committee to watch the Bill as a most important one. The voluntary hospitals should be willing to receive money from any body, local or central, but there should be a proviso as to the proportion such contribution bore to the total expense of the institution. The wounded men had been taken completely

(Continued on page iv.)

